# Effects of Endoscopic Submucosal Excavation With Non-Submucosal Injection on Stromal Tumors in Stomach

**DOI:** 10.3389/fonc.2022.792445

**Published:** 2022-04-04

**Authors:** Liang Huang, Yi-Xin Jia, Bin Lyu, Li-Na Meng, Hai-Feng Jin

**Affiliations:** Division of Gastroenterology, The First Affiliated Hospital of Zhejiang Chinese Medical University, Hangzhou, China

**Keywords:** endoscopic submucosal excavation (ESE), gastrointestinal stromal tumors, endoscopic full-thickness resection (EFTR), efficacy and safety, stomach tumor

## Abstract

**Background and Aim:**

Endoscopic submucosal excavation (ESE) is commonly used to treat gastrointestinal stromal tumors (GISTs), especially for tumor sizes within 2 cm; compared with the conventical ESE, the efficacy and safety of the no-submucosal injection (NSI) ESE remains unclear. The aim of this study was to assess the clinical efficacy and safety of NSI-ESE for gastric stromal tumors.

**Methods:**

ESE was performed in 102 patients at our hospital between January 2018 and January 2020, and the clinical features, surgical outcomes, complications, cost of performance, pathological diagnosis, and risk classification were evaluated.

**Results:**

All tumors were completely resected by endoscopic resection (ER), with a complete resection rate of 100%. It was achieved by ESE/EFTR (endoscopic full-thickness resection) in 49 cases with submucosal injection, and by ESE/EFTR in 53 cases with NSI-ESE. The mean surgical time in cases with submucosal injection was 25.86 ± 4.45 min, compared to the cases without submucosal injection (17.23 ± 3.47 min), and the difference was significant (*p* < 0.001); the exposure time of tumor, the time of complete excavation of tumor, procedure cost, and hospital stay in the NSI-ESE group were all lower than those cases with submucosal injection (*p* < 0.05). In the risk classification, 95 (93.1%) cases had a very low risk, 4 (4.0%) cases had a low risk, and 2 (2.0%) cases had a high risk. No recurrence or metastasis was observed during the follow-up period of 18 ± 6 months (range: 13–25 months).

**Conclusions:**

NSI-ESE is a feasible, effective, and safe treatment for gastric GISTs; compared to the conventional ESE, NSI-ESE has the following advantages: it decreases procedure time, it lowers the risk of perforation, and it is cost-effective.

## Introduction

Gastrointestinal stromal tumors (GISTs) are common in the stomach (60%–70%), and most primary GISTs are benign, but they have the tendency to become malignant as they increase in size (1, 2). In the past, for GISTs without metastasis, many scholars believed that the combination of surgery and laparoscopy is the best choice, but with the development of endoscopic technology, this concept is gradually changing. According to the National Comprehensive Cancer Network, surgical therapy is recommended for GISTs larger than 2 cm, and either surgical removal or surveillance is advised for those smaller than 2 cm (3). Recently, it has been indicated that even small GISTs (<2.0 cm) with a high mitotic index are also potentially malignant (4, 5), and some patients cannot deal with long-term survival with tumors because of the huge psychological and economic burden. Thus, it becomes more and more acceptable to diagnose and treat GISTs at an early stage as long as the patients are willing.

Endoscopic therapy, such as ESE (endoscopic submucosal excavation), EFTR (endoscopic full-thickness resection), and STER (submucosal tunneling endoscopic resection), has a great advantage in treating GISTs (6). The conventional procedural steps of those methods all involve submucosal injection to depart the mucosal from muscularis propria, but we found that the procedure has encountered some clinical problems: the purpose of submucosal injection is to separate the tumor from the surrounding tissue as much as possible, so that it may be easy to remove. However, we often find that the tumor may shift with the lifting of the mucosa after injection, so sometimes it takes more time to find the tumor, which may increase the risk of bleeding and perforation, especially for tumors less than 1 cm in size. However, can we perform no-submucosal injection (NSI) endoscopic resection (ER) for GISTs in the stomach? It is uncertain and has no related published research thus far. This study is focused on determining the feasibility, efficacy, and safety of no-submucosal injection ESE/ESFR in gastric stromal tumors in the stomach.

## Patients and Methods

### Subjects

The clinical data of 102 patients who accepted the conventional ESE/ESFR or NSI- ESE/ESFR at the Affiliated Hospital of Zhejiang Chinese Medical University (Hangzhou, China) between 2018 and 2020 were retrospectively analyzed. All patients received ultrasonography and/or CT scanning of the abdomen before surgery and EUS (endoscopic ultrasonography) was performed to assess the layer of origin and the exact tumor size.

### Endoscopic Procedures

**Figure 1:**
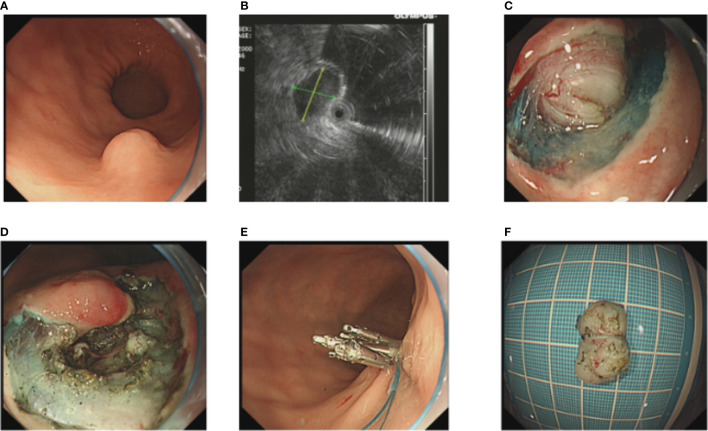
The conventional endoscopic submucosal dissection of a gastric GIST. **(A)** A gastric GIST is observed. **(B)** The tumor originates from the muscularis propria layer on EUS. **(C, D)** After submucosal injection, endoscopic submucosal excavation of the tumor is performed using a Hook knife and the lesion is removed completely. **(E)** The gastric wall defect was managed by clips and a nylon rope. **(F)** View of the tumor after resection.

**Figure 2:**
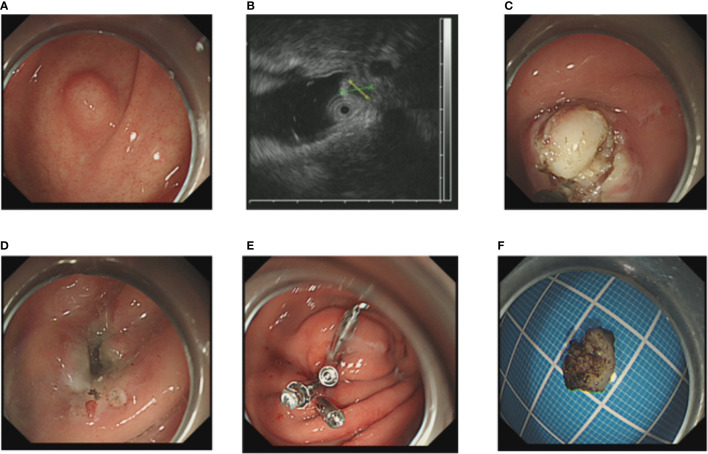
The NIE endoscopic submucosal dissection of a gastric GIST. **(A)** A gastric GIST is observed. **(B)** The tumor originates from the muscularis propria layer on EUS. **(C, D)** Without submucosal injection, endoscopic submucosal excavation of the tumor is directly performed using a Hook knife and the lesion is removed completely. **(E)** The gastric wall defect was managed by clips. **(F)** View of the tumor after resection.

After performance of EUS (GF-EU260, Olympus Co., Ltd.), the endoscopic procedures were performed by two experienced doctors (BL and H-FJ) using a gastroscope (GIF-Q260J; Olympus Co., Ltd.). Propofol was infused for anesthesia, and the patient was kept consciously sedated with cardiorespiratory monitoring during surgery. The conventional ER procedures were as follows (**Figure 1**): First, argon plasma coagulation was used for marking at 3–5 mm from the tumor margin, then an appropriate dose of indigo carmine (0.2%) was injected, which was added to 0.9% normal saline into the MP layer. Secondly, a Hook knife (KD-620LR, Olympus Co., Ltd.) was used to make a small incision to the submucosa, then the tumor was completely dissected along the lower edge of the tumor and above the muscularis propria by an insulation-tipped (IT, KD-611L, Olympus Co., Ltd.) knife or Hook knife. During the procedure, bleeding was managed successfully with hot biopsy forceps. Metal clips [ROCC-D-26-195-C, Micro-Tech (Nanjing) Co., Ltd.] were used for closing the wound and perforation. Several lesions were completely resected, including the serosal layer, and the gastric wall defect was managed by clips or a nylon rope (Le Clamp, China), as shown in **Figure 1**. The non-submucosal injection ER procedures were as as shown in **Figure 2**. First, a certain incision length (a little longer than the size of the tumor) at the edge of the tumor (usually at the vertex of gravity) was made to the submucosa directly with a Hook knife without submucosal injection and then en bloc resection of the tumor was achieved.

### Histopathological Evaluation

The removed tumors were subjected to formalin (10%) fixation immediately after the endoscopic procedure. CD34, CD117, S-100, SMA, Ki-67, and DOG-1 were detected using immunohistochemical staining. The mitotic index was calculated under 50 HPF (high-power fields), and the risk classification standard of GISTs was according to the National Institutes of Health (7).

### Follow-Up

The patients were followed up by gastroscopy 6 months after endoscopic therapy and annually thereafter by gastroscopy, and the abdomen was scanned to observe wound healing and exclude any tumor recurrence or residues.

### Statistical Analysis

The analyst was blinded to the study. SPSS version 22.0 statistics software (SPSS Inc., Chicago, IL, USA) was used to analyze data, and the measurement data variables were expressed as mean ± standard deviation. *t*-test was used for normal distribution quantitative data, and Wilcoxon Mann–Whitney rank sum test was used for non-normal distribution quantitative data. The number and percentage of use cases of counting data are expressed using chi-square (*χ* ^2^) test or the Fisher exact probability method, and the difference is considered to be statistically significant at *p* < 0.05.

## Results

### Clinical Characteristics

There were 53 patients who received non-submucosal injection ER, namely, 15 men and 38 women aged from 21 to 73 years (average age was 41.29 years), and there were 49 patients who received the conventional ER, namely, 14 men and 35 women aged from 26 to 72 years (average age was 45.37 years). There were 78 patients (75%) having clinical symptoms, the most common being upper abdominal discomfort. The others were found by regular physical examination. Of the 102 cases, the number of gastric stromal tumor located at the cardia is 10; gastric fundus, 51; gastric corpus, 27; and antrum, 14. All tumors originated from the MP according to the EUS findings. Metastasis was absent in all patients. The clinicopathological features of the patients are listed in **Table 1**.

**Table 1:** Characteristics of the patients and GISTs.

	Total	The conventional group	The NSI group	*p*-value
Age (years) (mean ± SD)	56.82 ± 7.89	56.87 ± 5.56	56.78 ± 4.25	0.536
Gender, *n* (%)				0.479
Male	29 (28.4)	14 (37.4)	15 (28.3)	
Female	73 (71.6)	35 (62.6)	38 (71.7)	
Symptomatic, *n* (%)	80 (78.4)	38 (77.6)	42 (79.2)	0.328
Asymptomatic, *n* (%)	22 (21.6)	11 (22.4)	11 (20.8)	0.967
Tumor site, *n* (%)				0.465
Gastric cardia	10 (9.8)	5 (10.2)	5 (9.5)	
Gastric fundus	51 (50)	24 (49)	27 (50.9)	
Gastric corpus	27 (26.5)	14 (28.6)	13 (24.5)	
Gastric antrum	14 (13.7)	6 (12.2)	8 (15.1)	
Tumor size, *n* (%)				0.562
≤20 mm	93 (91.2)	44 (89.8)	49 (92.5)	
>20 mm	9 (8.8)	5 (10.2)	4 (7.5)	

p: The conventional group vs. the NSI group.

### Outcomes of ER

All GISTs were completely resected by ER, with a complete resection rate of 100%. It was achieved by ESE in 32 cases, by EFTR in 17 (34.7%) cases using conventional ER, by ESE in 32 cases, and by EFTR in 21 (39.6%) cases using non-submucosal injection ER. There was no tumor spillage or rupture. The mean surgical time in cases with submucosal injection was 25.86 ± 4.45 min, compared to the cases without submucosal injection (17.23 ± 3.47 min), and the difference is significant (*p* < 0.001), but the difference in EFTR rates between the two groups is not significant (*p* = 0.68). All the perforations were closed by clips or a nylon rope under the endoscope. The exposure time of tumor, the time of complete excavation of tumor, and procedure time without submucosal injection were all lower than those cases with submucosal injection, and the differences are significant (*p* < 0.05). Accordingly, the cost of procedure and the hospital stay were also lower than those cases with submucosal injection (*p* < 0.05) (**Table 2**).

**Table 2:** Outcomes of ER.

	Total	The conventional group	The NSI group	*p*-value
Performed ESD (%)	64	32	32	0.89
Performed EFTR (%)	38	17	21	0.68
Tumor exposure time	2.11 ± 0.82	2.73 ± 1.21	1.32 ± 0.55	<0.001
Time of complete excavation of tumor	20.25 ± 3.43	25.86 ± 4.45	17.23 ± 3.47	<0.001
Overall removal rate of tumor (%)	100	100	100	
Procedure time (min)	26.27 ± 5.28	28.90 ± 6.77	21.98 ± 5.65	<0.001
Delayed hemorrhage, *n* (%)	0 (0)	0 (0)	0 (0)	
Cost of procedure ($)	461.66 ± 19.32	480.42 ± 21.47	436.79 ± 18.96	0.002
Hospital stays (days)	6.93 ± 1.34	7.26 ± 1.55	6.31 ± 1.42	0.03
Recurrence, *n* (%)	0 (0)	0 (0)	0 (0)	

p: The conventional group vs. the NSI group.

### Pathological Characteristics and Risk Classification

The mean tumor size was 1.09 ± 1.32 cm (range: 0.5–4.0 cm). The mitotic index in one patient was over 5 mitoses/50 HPF. The immunohistochemistry indicated that CD117 was positive in 85 patients (83.3%), CD34 was positive in 93 (91.2.5%), DOG-1 was positive in 85 (83.3%), and SMA was positive in 63 (61.8%) patients. The labeling index (LI, %) of Ki-67 was less than 5% in each case. In the risk classification, 95 (93.1%) had a very low risk, 5 (4.9%) had a low risk, and 2 (2.0%) had a high risk.

### Characteristics of EFTR Cases

Among these cases (38), there were 7 (18.4%) tumors that were located at the gastric cardia, 23 (60.5%) at the gastric fundus, 6 (15.8%) at the corpus, and 2 (5.3%) at the antrum. The differences in time of tumor exposure, the time of complete excavation of tumor, and time of procedure between the two groups are significant (*p* < 0.05) (**Table 3**); the EFTR without submucosal injection proved to be more efficient than the conventional EFTR.

**Table 3:** Characteristics of EFTR cases.

	The conventional group	The NSI group	*p*-value
Performed EFTR, *n*	17	21	
Tumor site, *n* (%)			0.578
Gastric cardia	3 (17.6)	4 (19.0)	
Gastric fundus	10 (58.2)	13 (61.9)	
Gastric corpus	3 (17.6)	3 (14.3)	
Gastric antrum	1 (5.9)	1 (4.8)	
Accidental perforation, *n*	5	0	<0.001
Tumor size, *n* (%)			
≤20 mm	15 (88.2)	20 (95.2)	
>20 mm	2 (11.8)	1 (4.8)	
Tumor exposure time	3.85 ± 1.45	1.47 ± 0.87	0.005
Time of complete excavation of tumor	31.33 ± 6.28	18.13 ± 2.55	<0.001
Procedure time (min)	36.37 ± 9.43	26.34 ± 6.87	0.02

### Follow-Up

All patients were followed up for more than 6 months (18 ± 6 months). Abdominal ultrasonography/scan and gastroscopy were performed in each patient, and there was no recurrence, metastasis, or death during the follow-up period.

## Discussion

Patients with GISTs have always been treated by surgery (8). With the advancement in endoscopic technology, this situation is now changing. Compared to surgery, endoscopic therapy not only has great advantages in terms of surgical time, postoperative recovery, and cost of surgical treatment (9), but also can preserve most structures of the stomach with normal digestive physiology maintained, resulting in a better quality of life for patients (10, 11). ESE and EFTR enable deep excavation, which is suitable for stomal tumors; compared with surgery, patients experience almost the same treatment effect but with a lower incidence of adverse events and trauma (12). Of course, for GISTs with highly suspected malignant tendency, surgical treatment is still the first choice. Compared to the published studies, in our study, there were 102 gastric stromal tumors removed using endoscopic therapy, namely, 64 by ESE and 38 by EFTR. The en bloc resection rate was 100%, and no one had to undergo surgery, demonstrating that ESE/EFTR is a safe and minimally invasive procedure for the removal of GISTs.

The most common complication of the use of ER for GISTs is perforation. According to previous studies, the incidence of perforation was 0%–22% (13, 14). In this study, it affected 38 patients (37.3%), including 33 intentional perforations and 5 accidental perforations in the conventional ESE group, which were closed immediately during the procedure (if the closing procedure makes the subsequent operation difficult, we will do it after removing the tumor). In EFTR, intentional perforation is not considered a complication. When the tumor originated from the deep muscularis propria layer and adhered tightly to the serosa, in order to remove the tumor completely, EFTR should be a better choice (15). We analyzed the data of EFTR patients and found that there were 17 patients in the conventional group and 21 patients in the NSI group. Overall, the most common location of perforation was gastric fundus (60.5%), followed by gastric cardia (18.4%), and it seemed to have no relationship with the size of tumor (compared with the other tumors, the difference in size was not significant, *p* > 0.05). According to published studies, perforations more commonly occurred while treating GISTs, because most stromal tumors are derived from deep within the muscularis propria and were tightly adherent to the surrounding tissue (16). In our study, we observed those tumors removed by EFTR and found that all of them originated from the deeper MP layer and had tight and wide adherence to muscle fibers; in some cases, there was even no MP layer left after the removal of tumors. Our study indicated that the difficulty of the ESE/EFTR procedure has a close relationship with the area of the tumor in the MP layer. Bialek et al. (17) also pointed out that complete tumor removal was only related to an absent or narrow connection of tumors with the MP layer. In this study, all of the wound surfaces in the case of “intentional” perforation were completely closed by clips or a nylon band together with clips under endoscopy after removing the tumor, and some clinical indexes including length of hospital stay and procedure cost were almost the same as other patients. Thus, similar to published studies, our study also showed that EFTR is a safe and feasible option for GISTs (18).

However, there are some problems encountered in the use of conventional ER, especially in ESE treating small-sized GISTs. According to the conventional ER, we always give a submucosal injection of 0.9% normal saline with an appropriate dose of indigo carmine and epinephrine in order to separate the tumor from other tissues before incision of mucosa, but we have found that there are some disadvantages here: first, we sometimes have to spend more time finding the tumor because the location of the tumor may change after submucosal injection, especially when the tumor is less than 1 cm in size, and it is not easy for us to distinguish normal muscularis propria from tumors covered with muscularis propria, so the risk of accidental perforation may increase. As shown in **Table 3**, there were 5 patients who had accidental perforation in the conventional group because of the unsure location of the tumor after submucosal injection, and there was no accidental perforation in the NSI group. Of course, due to the small sample size and non-multicenter research and other factors, we cannot hastily conclude that the probability of accidental perforation using conventional ESD methods is certainly higher than that of non-submucosal injection ESD methods. Consistent with this is the tumor exposure time, which is 1.47 ± 0.87 min in the NSI group, shorter than that in the conventional group (3.85 ± 1.45 min, *p* = 0.005), which showed that we could find the tumor faster by making a direct incision near the tumor without submucosal injection; accordingly, the time of complete excavation of tumor and procedure time were also shorter than the conventional group (*p* < 0.05), and there was no other complication in the NSI group. These data showed that the NSI ESE is a safe and efficient method for treating GISTs.

At present, many national medical insurance companies adopt a payment mode closely related to diagnosis-related groups (DRGs), which requires clinicians to solve clinical problems for patients with the most cost-effective means (19, 20). Our study’s data showed not only that the NSI ESE is a safe and efficient method for the treatment of GISTs, but also that it is a cost-effective treatment. As shown in **Table 2**, the cost of procedure and hospital stay in the NSI group was lower than that of the conventional group, which might be due to the non-use of injection needles and shorter procedure time, which may have a relationship with recovery from the procedure. Furthermore, the more than 1 year-follow up showed that the recurrence in both groups was 0, indicating that the NSI ESE may potentially be a better treatment for GISTs.

The pathological risk is a very important factor for GISTs, because it has a close relationship to the follow-up treatment plan and even related to the prognosis of patients (21). According to our study, most patients had a very low risk, and only two cases had a high risk based on the mitotic index and tumor size. The two patients were given imatinib mesylate to prevent metastasis or recurrence. During the follow-up period of 14 months, there was no tumor recurrence and metastasis. It was reported that survival of gastric GIST patients who had Ki-67 LI ≥ 5% was shorter compared to those with Ki-67 LI < 5% (22); in our study, all 102 patients with GISTs had Ki-67 LI < 5%.

There are several limitations in this study. Firstly, this is a retrospective study and may have some impact on the research results. Secondly, the sample size is not very large, and a single-center study remains a shortcoming.

In conclusion, as far as we know, this is the first study to observe the clinical possibility of no-submucosal injection ESE and its advantages in treating GISTs compared to the conventional ESE, and the results showed that NSI-ESE is a feasible, effective, and safe treatment for gastric GISTs. Compared to the conventional ESE, NSI ESE has the following advantages: it decreases procedure time, it lowers the risk of perforation, and it is cost-effective. Of course, the efficacy and safety of NSI ESE in gastric stromal tumor need to be further investigated by future prospective multicenter studies.

## Data Availability Statement

The original contributions presented in the study are included in the article/supplementary material. Further inquiries can be directed to the corresponding author.

## Ethics Statement

The studies involving human participants were reviewed and approved by the ethics committee of First Affiliated Hospital of Zhejiang Chinese Medical University. The patients/participants provided their written informed consent to participate in this study.

## Author Contributions

H-FJ and LH performed the whole experiment, statistical analysis, and wrote the manuscript together. Y-XJ and L-NM participated in the experiment. BL designed the study. All authors contributed to the article and approved the submitted version.

## Funding

This work was supported by the Project of Zhejiang Medical and Health Science and Technology Plan (2019PY052 and 2017KY512), the Project of Zhejiang Traditional Chinese Medicine Science and Technology (2017ZKL008 and 2016za092), and the Key Laboratory of Pathology and Physiology of Digestive Tract Disease in Zhejiang Province.

## Conflict of Interest

The authors declare that the research was conducted in the absence of any commercial or financial relationships that could be construed as a potential conflict of interest.

## Publisher’s Note

All claims expressed in this article are solely those of the authors and do not necessarily represent those of their affiliated organizations, or those of the publisher, the editors and the reviewers. Any product that may be evaluated in this article, or claim that may be made by its manufacturer, is not guaranteed or endorsed by the publisher.
